# Epigenetic Regulation of Epidermal Stem Cell Biomarkers and Their Role in Wound Healing

**DOI:** 10.3390/ijms17010016

**Published:** 2015-12-24

**Authors:** Sabita N. Saldanha, Kendra J. Royston, Neha Udayakumar, Trygve O. Tollefsbol

**Affiliations:** 1 Department of Biological Sciences, Alabama State University, Montgomery, AL 36104, USA; 2 Department of Biology, University of Alabama at Birmingham, Birmingham, AL 35294-1170, USA; kendroy@uab.edu (K.J.R.); nehau@uab.edu (N.U.); trygve@uab.edu (T.O.T.); 3 Clinical Nutrition Research Center, University of Alabama at Birmingham, Birmingham, AL 35294-1170, USA; 4 Comprehensive Cancer Center, University of Alabama at Birmingham, Birmingham, AL 35294-1170, USA; 5 Center for Aging, University of Alabama at Birmingham, Birmingham, AL 35294-1170, USA; 6 Nutrition Obesity Research Center, University of Alabama at Birmingham, Birmingham, AL 35294-1170, USA

**Keywords:** epigenetics, wound healing, skin, microRNA, epidermal stem cells, biomarkers

## Abstract

As an actively renewable tissue, changes in skin architecture are subjected to the regulation of stem cells that maintain the population of cells responsible for the formation of epidermal layers. Stems cells retain their self-renewal property and express biomarkers that are unique to this population. However, differential regulation of the biomarkers can initiate the pathway of terminal cell differentiation. Although, pockets of non-clarity in stem cell maintenance and differentiation in skin still exist, the influence of epigenetics in epidermal stem cell functions and differentiation in skin homeostasis and wound healing is clearly evident. The focus of this review is to discuss the epigenetic regulation of confirmed and probable epidermal stem cell biomarkers in epidermal stratification of normal skin and in diseased states. The role of epigenetics in wound healing, especially in diseased states of diabetes and cancer, will also be conveyed.

## 1. Introduction

**Figure 1:**
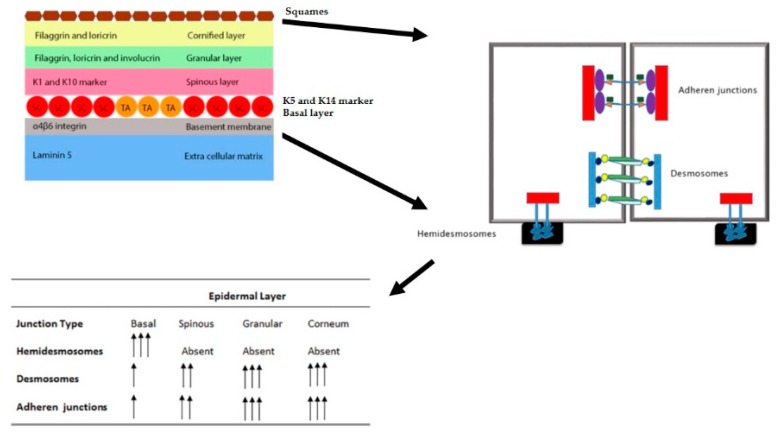
Epidermal stem cell biomarkers and epidermal stratification. Epidermal stratification of skin is a finely orchestrated process that maintains skin architecture as well as homeostasis. Epidermal stem cells are found at specific locations in the skin and specifically those found in the interfollicular epithelium (IFE), and basal cells undergo division to give rise to a population of cells with lineage commitment. The primary division of epidermal stem cells (ESCs) give rise to two sets of cells; one that retains ESC sternness and the other with limited division and lineage commitment called transit amplifying (TA) cells. TA cells after a few rounds of division produce cells that are destined to form the supra basal layers. Based on the cell fate destination, the TA originated cells express markers specific to epidermal layer occupancy. Such that cells expressing K5 and K14 biomarkers occupy the immediate first layer above the basal layer and consist of cells with a spinous membranous structures. Further, as the cells get pushed upward, the expression of K1 and K10 biomarkers place cells in the granular layer with deposition of granules inside the cells. The induction of filaggrin and loricrin along with keratin create cellular condensation and layer compaction contributing to the cornification of the stratum corneum. As cells are pushed from the basal layer to the top of the epidermis, the number as well as the type of junctions change mediating keratinocyte flexibility and plasticity and tissue integrity. In the basal layer, the number of adheren junctions are fewer and hemidesmosomes are more. Such a framework provides the possibility for keratinocyte migration and epidermal turnover. The basement membrane (BM) is tethered to the extra cellular matrix (ECM) by integrin molecules in conjunction with laminin 5 of the ECM matrix and mediate basal cell stability with the BM through integrin-keratin connections. As the cells move upward both desmosome and adheren junctions increase and their connection to intracellular keratin further strengthens the tissue architecture. As shown in the figure, the following symbols represent the respective epidermal markers and ECM proteins. Red rectangle, actin; green small square, p120; orange triangle, β-catenin; purple oval; α-catenin, irregular blue shape, E-cadherin; elongated blue rectangle, keratin; irregular dark green shape, desmoglein; irregular light green shape, desmocollin; dark blue circle, desmoplqakins; yellow circle plakoglobin; squiggle blue shape, integrin and black rectangle, basement membrane. SC = Stem cells and TA = transit amplifying cells. The arrows in the table indicate the number of specific-type of junctions in the respective epidermal layers. A single arrow indicates the presence of very few specific-type of junctions; a double arrow indicates a few more and triple arrows indicate an even greater presence of these junctions in the respective epidermal layers.

The adult skin consists of an array of organized layers critical to dermal functions. Stem cells residing in the epidermal layer allow for the homeostatic maintenance of adult skin and includes skin replenishment, cornification and sloughing off of old skin, hair generation, and tissue repair after injury [1]. Different types of stem cells enable the homeostatic functions of the skin, among which melanoblasts and epidermal stem cells (ESCs) are present in the skin itself [1,2]. ESCs are multipotent and are committed to the formation and differentiation of the epidermis in normal skin formation and during the healing process of the skin as well [3]. Structurally, in skin, the underlying dermis is separated by a basement membrane overlaid by a stratified epidermis. Epidermal stratification is a continuous process that begins during embryonal development and is maintained throughout life [4]. Keratinocytes are the most abundant cell population of the epidermis and express intermediate filaments, namely keratins [5]. Dermal stratification is regulated by ESCs and the regulation of biomarkers expressed by the cells control the commitment and differentiation of the keratinocytes. Thus, the progression of commitment begins with a basal cell differentiating to a spinous cell followed by a granular cell and terminating into a differentiated enucleated cornified keratinocyte [6] (Figure 1; Table 1).

Stem cells (SCs) of the epidermis exits in specific pockets known as bulges and are found in the hair follicle, interfollicular epithelium (IFE) and sebaceous glands [1] (Table 1). Identification of ESCs in these regions have been supported by population enrichment studies in murine models, where slow-cycling stem cells are screened from other cells based on label retention of bromodeoxyuridine or [3H] thymidine. Similarly, commitment of the SC lineage has been assessed through transgenic cell lines expressing green fluorescent protein (GFP) under the regulation of the keratins *K5* and *K15* promoter [1,7].

Isolation of SCs through this method showed that the cells were capable of giving rise to all types of epidermal cells of the skin [8]. Unlike the SCs in the bulges that have high multipotent potential and self-renewable capabilities, SCs residing in the basal layer of the IFE are less multipotent and effective giving rise to committed and terminally differentiated lineages after a few rounds of cell division [9]. These cells arising from basal cells with limited self-renewable potential but lineage specification are transit amplifying (TA) cells [10].

**Table 1:** Cell type distribution in epidermal layers.

Skin Layer	Intermediate Layers/Structures	Cell Populations Found	Type of Epidermal Stem Populations	Phenotypic Output of the Stem Cells	References
Epidermis	Basal layer	Undifferentiated cells	Interfollicular (IF)	IF epidermis	[1]
	Spinous layer	Partially differentiated	–	–	[1]
	Granular layer	Partially differentiated	–	–	[1]
	Cornified layer	Terminally differentiated	–	–	[1]
	Bulge	Undifferentiated cells	Hair follicle stem cells (HFSC)	Hair follicle	[11]
	Sebaceous gland	Undifferentiated cells	sebaceous gland (SG) stem cells	Sebaceous gland	[12]

The extracellular matrix (ECM) materials produced by cells of the basal layer contain laminin 5 and integrins that separate the epidermal structures from the dermis (Figure 1). Heterodimeric keratins connect to α6β4-integrin anchoring the basement membrane of the epidermis to the ECM. Keratin filaments also tether intercellular junctions called desmosomes via cadherins and together provide an extensive framework to the epithelium [13]. Desmosomes are more abundantly present and associated with suprabasal cells than with basal cells, and the association of basal cells with cytoskeletal filaments through adherent junctions, involving α and β catenins with E-cadherin and αβ1-integrin cell-ECM junctions, contribute to epidermal stability, which is lost in cancer [14]. Filaggrin and loricrin expression are observed in the granular layer [15]. Filaggrin is a 37 KDa protein that is expressed in terminally differentiated keratinocytes and connects to keratin filaments condensing the keratin cytoskeletal framework for cellular compaction necessary for squame biogenesis [16]. Terminal differentiation of keratinocytes is orchestrated by several transcription factors which include activator protein 1 (AP1), activator protein 2 (AP2), CCAAT/enhancer binding proteins (C/EBPs), Krüppel-like family of transcription factors (Klfs), Peroxisome proliferator-activated receptors (PPARs), and Notch [1].

Epigenetic changes are heritable yet reversible and are fundamentally regulated by three major epigenetic mechanisms: DNA methylation, histone modifications and microRNAs (miRNAs) [17,18]. The role of DNA methylation and histone modifications in skin homeostasis and wound healing are just beginning to emerge and are discussed in detail in other reviews [19]. Overall, studies focusing on the epigenetic regulation of ESCs biomarkers are rather limited and the role of epigenetic mechanisms in the regulation of the biomarkers is not well understood due to the paucity of research in this area. However, the few studies that have evaluated epigenetic influence on the expression of ESC biomarkers have shown that miRNAs play a prominent role in the regulation of these biomarkers which affect the epidermal stratification process. The primary focus of this review is to discuss the regulation of ESC biomarkers in epidermal stratification. Further, the role of the ESC biomarkers in wound healing and the effects on tissue repair and skin homeostasis will also be addressed.

## 2. Epidermal Stem Cells and Biomarkers

The skin tissue consists of stratified squamous epithelial layers, of which the innermost basal layer retains proliferative potential. The three populations of epidermal stems cells found in the basal layer of the epidermis are classified as holo, para and metaclones [20]. The holoclones express β1-integrin, α6-integrin and low levels of CD71 (transferrin receptor) [21]. These are quiescence and slow-growing cell populations with self-renewable capabilities. *In vitro* experiments have shown that the cells retain-label and therefore correspond to the stem cell population and localize in the downward tip of the rete ridges [20]. Cells that are β1-integrin^+^/melanoma chondroitin sulfate proteoglycan (MCSP)^+^/leucine-rich α-2-glycoprotein 1 (Lrg1)^+^, are present in the upper segment of the rete ridge [20]. Survivin is another biomarker for these populations. Paraclones, give rise to colonies of cells that differentiate after limited proliferation and are classified as TA cells [20]. Other biomarkers responsible for changes in ESC to differentiated keratinocytes are discussed in the following sections (Figure 1).

### 2.1. Integrins

The basal layer secrets ECM, predominantly laminin 5 and uses α3β1-integrin for assembly [22]. The ECM layer separates the epidermis from the dermis and serves as a point of anchorage of basal cells to the basement membrane tethered by ECM filaments [23,24]. As cells from the basal layer move outwards towards the surface, they withdraw from the cell cycle, transcriptionally silence integrin and laminin expression, and induce terminal differentiation [1]. However, in the production of the intermediate spinous and granular layers, these proteins remain expressed but at the surface are switched off and culminate in the production of squames that are sloughed from the skin surface and replaced by inner cells moving outward [1]. β1 and α6β4 integrins are part of the ECM architecture and have putative structural and regulatory roles in various cell types, including the epidermis [14]. Integrins relay signals from the ECM to cells and help with cyctoskeletal organization that is important for proliferation, apoptosis and differentiation [14]. A study by Li *et al.* showed that the combination of high levels of α6 integrin and low levels of transferrin protein is associated with ESCs characterized by proliferative potential [25]. However, findings from other investigations do not support this observation as the presence of α6β4 integrins or not does not affect epidermal proliferative capacities [26]. β1 integrin is expressed throughout the basal layer of the epidermis and is necessary for maintaining keratinocytes in the non-differentiated state [26]. The down-regulation of this biomarker is observed in keratinocytes marked for terminal differentiation and is supported by studies showing that conditional knockout of this protein induces aberrations in epidermal proliferation and basement membrane formation [22]. Nonetheless, its relevance as a SC biomarker still remains in question.

### 2.2. Cadherins/Catenins

Both epithelial (E)- and placental (P)-cadherins regulate important processes in development, in addition to mediating cell–cell adhesion functions [27,28]. Cadherins are crucial components of adheren junctions (AJ) and their connections with catenin and keratin molecules enhance and maintain dermal integrity which is often lost in tumor development and metastasis [29]. Deregulation of E- and P- cadherins contributes to skin disorders, enhanced cellular migration and invasion in tumor metastasis, and in the de-differentiation process [27]. The expression of these adheren molecules are regulated both by genetic and epigenetic events which will be discussed in the subsequent sections of the review. Through knockout studies, Tinkle and colleagues were able to show that the levels rather than cadherin subtype were critical to keratinocyte stability and thus epidermal integrity [29]. Loss of epidermal integrity due to cadherin insufficiency lead to defects similar as seen in mice mutant for α-catenin [29]. Keratinocyte plasticity, flexibility and ease of migration is governed by adheren junctions in conjunction with desmosomes and hemidesmosomes (Figure 1). Association of cadherins with catenin components is essential to the formation of the adheren junction, and albeit, as individual units, they exhibit different proliferation and inflammatory responses, and in cohesion govern keratinocyte adhesion [29,30].

Intracellularly, in keratinocytes, the cadherin/catenine complexes of adheren junctions associate with the actin filaments mediating junctional stability through enhanced adhesion [31]. E-cadherins relay extracellular signals via its transmembrane component through Ca^2+^ binding [32]. The intracellular domain of E-cadherin binds to catenins (p120 and β) and then complexes with α-catenin [31,32,33]. Mutations in cadherin/catenin complex units increase the risk of cancer development and have also been known to contribute to skin cancers [27,30]. The loss of catenins destabilizes the AJ complex, enhances inflammation, proliferation and migration, initiating tumor pathogenesis [34].

Determination of keratinocyte populations in the basal layer of the epidermis have been performed using antibody-specific fluorescent staining. The resultant fluorescent intensities are then used to distinguish the population subtypes in the basal layer of the epidermis. Those cells that stain positive for β-1 integrin subunit and are seen as integrin-bright populations, constitute stem cells. Less intensity of staining are observed in cells with decreased proliferation capacities [35]. Besides β-1 integrin staining, a protein important to the adherence of basal keratinocytes to the basement membrane, antibody specific staining of AJ complex subunits reveal higher staining intensities for E-cadherin, β-catenin, and γ-catenin [36,37,38]. Thus, the levels of E-cadherin and of β- and γ-catenin with integrin may provide biomarkers for the stem cell compartment of basal keratinocytes [38].

### 2.3. Keratins

Keratins in conjunction with catenins and cadherins maintain the structural integrity of the epidermis [39]. Keratins are variously expressed in the epidermal stratified layers. The expression of K5 and K14 is seen in basal cells; K1 and K10 in spinous layers and filaggrin, loricrin and involucrin in the granular layer [40]. In basal keratinocytes, expression of keratin K5/K14 heterodimers are found to be associated with the expression of K15, and the expression of K15 in regulating stemness is associated with Forkhead box protein M1 (FOXM1) [41]. Conversely, K15 expression is also found in abnormal keratinocytes, and in cells of other tissues exposed to hormones and cytokines [42]. In the bulge of hair follicles, a predominant niche for stem cells, K15 expression is observed which has been supported by a few studies, thus implicating its potential as a stem cell marker [42]. Contradictory to its potential as a stem cell biomarker, K15 expression is also observed in suprabasal layers containing cells destined for differentiation, and the expression is tethered to the down-regulation of β1-integrin and the protein kinase C and activator protein-1 (PKC/AP-1) pathway [41]. Due to such contradictory findings, one must lean toward the side of caution regarding the suitability of K15 as a stem cell biomarker [41,42,43].

### 2.4. p63

p63 is an important transcription factor with pivotal functions in epidermal cell lineage commitment, and the absence of the protein in embryonal development negates epithelial stratification [44,45]. p63 is transcriptionally regulated by two promoters, *TA* and *δN*, each responsible for the expression of or lack of the N-terminal transactivating domain, respectively. The δN-p63 isoform, δN-p63-α is predominantly expressed in the basal layer and is required to maintain keratinocytes in the non-differentiated state [41,46,47]. TA-p63 isoforms are necessary for keratinocyte commitment and initiation of stratification of the epithelial layers and these functions are blocked by δpN-p63-α [45,48]. Since few investigations have been conducted to determine the functional significance of the p63 isoforms in epidermal stratification, it limits the understanding of the relevance of each isoform with respect to cell lineage determination and differentiation. Further studies are therefore warranted to provide critical data that determines the significant influence of these isoforms as cues in ESC stratification.

## 3. Epigenetic Regulation of Epidermal Stem Cell Biomarkers by miRNA Regulation

**Figure 2:**
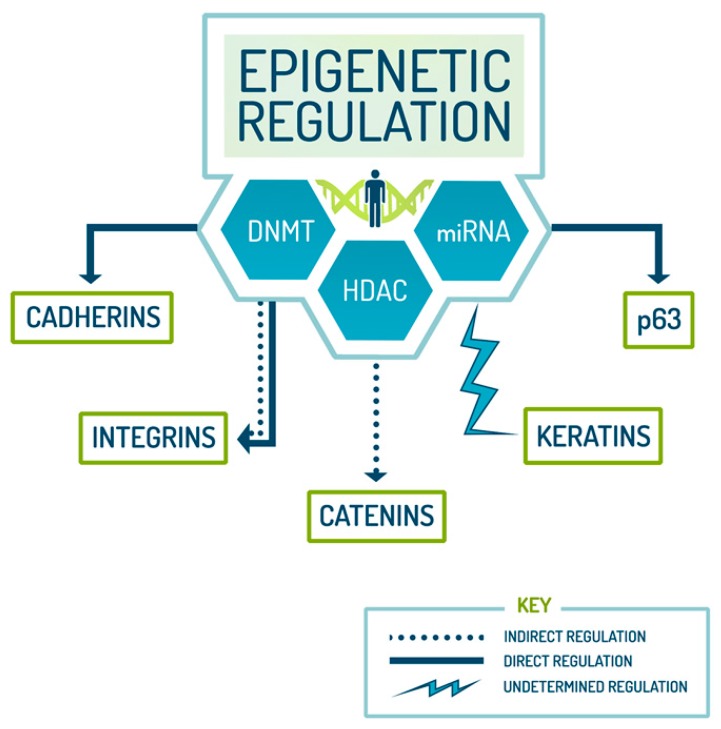
Epigenetic regulation of ESC biomarkers. Epigenetics has several roles in various processes. This image is a depiction of the relationship between epigenetic modifiers and ESC biomarkers. As indicated by the arrows, these modifiers directly regulate cadherins and p63, as well as indirectly regulate catenins. Note that integrins are both directly and indirectly modulated by epigenetics. As with keratins, an epigenetic relationship has been established. However, it still remains to be ascertained whether the relationship is of a direct or indirect nature and whether the resultant epigenetic modification fosters an overexpressed or inhibited phenotype of the protein. DNMT: DNA methyltransferase; HDAC: histone deacetylase.

The study of epigenetics encompasses an umbrella of cellular and molecular events associated with different areas of scientific research and includes modifications that regulate stem cells. This study of heritable changes in gene expression with no change to the underlying DNA sequence [49] has garnered much attention as traits that are epigenetically modified are typically reversible and relatively easily manipulated. Though the field of epigenetics has grown exponentially over the past decade, the lack of a complete understanding of all aspects of the field hinders the scientific community. As evident by the vast majority of the literature, epigenetic regulators such as histone deacetylases (HDACs) and DNA methyltransferases (DNMTs) are integral to several processes that control gene expression and, more recently, have been found to be associated with the regulation of epidermal stem cell biomarkers (Figure 2; Table 2). With current epigenetic technologies, substantial information on the individual roles of DNMTs and HDACs in stem cell differentiation, suppression of self-renewal and lineage commitment has been deciphered.

Since there are many papers that discuss HDACs and DNMTs in depth, here we will only provide a brief summary of their influence on the aforementioned biomarkers from the previous section.

**Table 2:** Epigenetic modifiers and their roles in stem cell regulation. DNMT: DNA methyltransferase; HDAC: histone deacetylase.

Epigenetic Regulation of Stem Cells		
Modifiers	Roles	References
miRNA	Stem cell differentiation and suppression of self-renewal, regulation of integrin, direct repression of p63	[50,51,52]
DNMT	Stem cell differentiation and lineage commitment, cadherin regulation, catenin management	[53,54,55]
HDAC	Cell division/chromosome segregation accuracy, and pluripotency, cadherin regulation, p63 repression	[19,54,56]

Studies reveal that both HDACs and DNMTs have some regulatory influence on cadherin, one of the ESC biomarkers responsible for cell adhesion [54]. In fact, DNMT inhibition by RG108, a DNA methyltransferase inhibitor, has been shown to enhance the expression of this biomarker [57]. Catenins, a biomarker that is required for the formation of the cadherin–catenin complex, is key in cadherin-mediated adhesion [58] and is regulated by DNA methylation [55]. Integrins, known for their roles as receptors for cell adhesion, are regulated epigenetically by HDACs and DNMTs [59]. An indication of abnormalities that lead to cancer is linked to the hypermethylation of certain keratins as recently reported by Naganuma and colleagues [60]. The hypermethylation-mediated epigenetic silencing of tumor suppressors and the inadvertent activation of oncogenes can be reversed by epigenetic therapy by using inhibitors specific to enzymes that target these processes. Lastly, the transcription factor p63, a master in the regulation of epidermal development [61], is also thought to be modified epigenetically. According to Botchkarev *et al.*, specific HDACs are key contributors to the upstream regulation of p63 [62].

MicroRNAs (miRNAs), more recent additions to the field of epigenetics, have also emerged as topics of extreme interest due to their posttranscriptional regulation of genes. Understanding the mechanistic actions of these small molecules and the pathways they regulate provides a means to discover new ways to approach the maintenance and elimination of life threatening diseases such as cancer. These non-coding RNAs are short and only about 22 nucleotides in length and are major players in translational repression by targeting mRNA [63,64]. One miRNA that has been studied in depth with respect to epidermal stem cells is microRNA-203 (miR-203). This non-coding RNA is considered to be a skin miRNA and is shown to have major roles in the promotion of differentiation through the repression of a specific ESC characteristic referred to as “stemness” [52]. Yi and colleagues, through their research, showed that p63 is directly repressed by miR-203 [52]. This finding is extremely intriguing; especially with p63’s major roles in the development of epidermal stem cells.

Song and associates found that the microRNA-9 (miR-9) represses cadherin and promotes the metastatic potential of esophageal squamous cell carcinoma, and induces epithelial mesenchymal transition. They observed that the knockdown of miR-9 resulted in the inhibition of migration and tumor metastasis [65]. From this information it can be inferred that miRNAs are major contributors to not only the regulation of ESC growth and development but also the aberrant signaling and mutagenesis of normal cells into cancerous ones. There are several miRNAs that have an effect on catenin. MicroRNA-200a (miR-200a) and microRNA-145 (miR-145) are known for their anti-tumor affects and have been recently shown to interact with catenin by suppressing the Wnt/β-catenin pathway which is instrumental in the progression of several different cancers [66,67]. It has also been discovered that microRNA-1826 (miR1826) is responsible for the direct down regulation of β-catenin in both renal and bladder cancer, as shown in 2012 by Hirata *et al.* [68,69].

Hepatoblastoma, a rare yet malignant type of liver cancer, is characterized by the over expression of pleomorphic adenoma gene 1 (*PLAG1*). von Frowein and colleagues performed a microarray analysis and identified miRNA-492 (miR-492) to be strongly influenced by PLAG1. They also provide evidence that miR-492 originates from keratin 19 suggesting a close functional relationship between the two [70]. Regulation of integrins by miRNA is widespread. In fact, several studies have provided evidence of multiple miRNAs both directly and indirectly repressing and regulating the signaling of integrins [71]. MicroRNA-149 (miR-149) is an example of one that targets a protein kinase receptor to indirectly suppress integrin signaling [72]. Another miRNA that has been studied is microRNA-378a (miR-378). Additional findings indicate that the upregulation of integrin β-3, which enhances wound healing capabilities in skin cells, is modulated by miR-378 [73].

Needless to say, the literature is ever evolving on the topic of epigenetics in regards to miRNAs. As more information becomes available, it is becoming extremely apparent that miRNAs are particularly instrumental in several areas of regulation, growth and development. They have the ability to control several biomarkers and processes that are responsible for phenotypic plasticity and a number of cellular functions. As research progresses, the importance of miRNAs is becoming apparent for they are involved in almost every organismal process and show reversible manipulation.

## 4. Epidermal Stem Cell (ESC) Biomarkers and Their Role in Wound Healing

Wound healing involves a complex orchestration of processes that suppress infection and restore the dermal barrier. Healing begins immediately following laceration to the epidermis with hemostasis, which includes initial vasoconstriction and thrombocyte clumping. This is followed by an influx of fibroblasts (which help clot the wound opening), and inflammatory macrophages. Subsequently, granulation tissue creates the framework for forming permanent connective tissue. This last stage involves of matrix fiber reconstruction, angiogenesis, and cell differentiation and proliferation, and epidermal remodeling [74,75]. The previously mentioned biomarkers are mainly involved in this last stage of the wound healing process.

### 4.1. Cadherins and Wound Healing

Cadherins are Ca^2+^-dependent, transmembrane glycoproteins that physically maintain cell-to-cell adhesion with cadherins on adjacent epithelial cells and withstand mechanical forces [74]. The cytoplasmic portion is connected to the biomarker β-catenin coupled to an α-catenin molecule that is directly attached to an intracellular actin filament [76]. Cadherin–catenin–actin complexes ensure mechanical adhesion of epithelial cells. During wound repair, E-cadherin alters cell adhesion to prepare for keratinocyte migration. Previous studies show that E-cadherin is down-regulated in basal epithelial cells surrounding the insult area during re-epithelialization [77]. It was found that when E-cadherin is not down-regulated by knocking out the epidermal regulatory gene (chicken ovalbumin upstream promoter transcription factor-interacting proteins 2 (*Ctip2*)), epithelial cells are more tightly packed together, thus inhibiting keratinocyte migration, and consequently, re-epithelialization itself [77]. Similarly, a recent study by Davids *et al.*, showed that E-cadherin expression promotes melanocyte migration into the wound area, as well as decreased keratinocyte attachment [78].

### 4.2. Integrins and Wound Healing

Integrins are heterodimeric transmembrane proteins that act as receptors to promote cellular adhesion. They are also heavily involved in bidirectional signaling, matrix assembly, apoptosis, transforming growth factor (TGF)-β1 signaling, and cytoskeleton organization and processes in normal and wounded tissues [79,80,81]. Regarding wound healing, integrins are mainly involved in the granulation and re-epithelialization stages. It was found in one study that β1-integrin null mice showed decreased cell migration and excessive hyperproliferation, supporting the claim that β1-integrins are necessary for re-epithelialization [74]. Integrin-coordinated ECM construction is vital to tissue remodeling after injury. Improper composition or mechanics of the matrix is related to chronic wound pathology [79]. Expression and relocalization of several integrins are induced in order to regulate epithelial migration and granulation. It was found that integrins α5β1 and α6β4 are most important for epithelial cell migration, whereas α5β1 regulates the fiber matrix of granulation tissue [82]. Additionally, β-3 integrins were also found to increase cell differentiation and fibroblast migration *in vivo* due to knockdown of miR-378a, a microRNA regulator of wound healing [73].

### 4.3. Catenins and Wound Healing

Both α-catenins and β-catenins are vital contributors in cadherin–catenin–actin complexes which maintain epithelial cell adhesion. While cadherin molecules are important in establishing extracellular connections between cells, catenin proteins form a bridge between the transmembrane cadherin and intracellular actin filaments [83]. Thus, they are responsible for uniting the intracellular and extracellular components of the cadherin junction. β-catenin is a key mediator of the Wnt signaling pathway, which is essential for several aspects of re-epithelialization during wound healing, especially the regeneration of hair follicles [84]. Wnt/β-catenin signaling activates hair follicle progenitor cells, which promote stem cell activity similar to embryonic development [84,85]. One study recently found that the chromatin effector Pygo2 helps β-catenin promote p53 activation in regenerating epidermal cells, although the exact mechanisms for this are still unknown [85]. Conversely, it was found that β-catenin and c-myc activation inhibits keratinocyte migration and differentiation and indirectly hinders cytokines and growth factors, therefore leading to the development of chronic wounds [86]. 

### 4.4. Keratins and Wound Healing

Keratin, an intermediate filament, is vital for establishing the durability of the cytoskeleton in keratinocytes. Keratin isotopes 6, 16, and 17 have been known to be upregulated around epidermal wound sites, and are essential for healing to take place [87]. In a 2012 study, it was found that both solid and liquid keratin wound dressings stimulated keratinocytes to re-epithelialize faster [88]. Keratin-based bandages are being used clinically and it is possible that the healing potential demonstrated in animal models can be seen in humans [89]. Moreover, very recently, Loschke *et al.* have indicated that some isotopes that are involved with maintaining desmosome adhesion (including keratins 1 and 10) are down-regulated during wound healing, whereas others, namely keratins 6, 16, and 17, are upregulated in keratinocytes [90]. There is great exigency for understanding the mechanistic role of keratin isotopes in wound healing to better the outcomes of patients who do not respond well to conventional wound healing procedures [89].

### 4.5. p63 and Wound Healing

The p63 protein is another transcript from the p53 family that regulates differentiation and proliferation of epidermal stem cells, and therefore is relevant when studying skin regeneration or tumorigenesis [91,92]. It was found that increased phosphorylated p63 levels in the wound area indicate that epidermal stem cells have differentiated into various progenitors, accompanied with reepithelialization of the skin tissue in mouse models, although details concerning p63 signaling pathways remain unknown [91]. A different study by Warner *et al.* shows that N-terminally truncated p63 levels are directly related to β-catenin expression and wound closure ability. In addition, the expression of E-cadherin was increased, which may indicate inhibited cytokinesis of epithelial cells [93]. Due to the roles of ESC biomarkers in wound healing, and their epigenetic regulation, it is safe to conclude that epigenetics is a vital part of wound healing and repair.

## 5. Epigenetic Regulation of Wound Healing in Normal State and Disease

Tissue repair in normal skin is dependent on the replicative potential of stem cells. Chronic wounds with increased inflammation predisposes tissues to tumor development that has been observed in skin carcinogenesis [94]. Although the mechanistic functions and regulation of epidermal stem cells in wound healing and cancer are not completely understood, investigations conducted thus far have shown that the deregulation of specific signaling pathways such as Wnt critical to stem cell behavior, normal keratinocyte differentiation and epidermal stratification are affected and responsible for tumor development [95]. Due to paucity of research in the epigenetic programs that regulate the wound healing process, the mechanistic implications of the epigenetic events in tissue repair are not quite clear and need to be unveiled. The question remains how and which SCs (basal layer, hair follicular bulge or IFE) are involved in wound healing [96]. Differential outcomes of stem cells in wound healing are governed by epigenetic mechanisms. These controls regulate cellular milieu which engineer the differentiation and commitment of stem cells to either epidermal cells or hair follicle cells in the wound healing process. Histone modifying enzymes, HDACs and histone methyltransferases (HMTs) are crucial for epidermal and hair follicular development. The absence of specific HMTs, enhancer of zeste 1 (Ezh1) and enhancer of zeste 2 (Ezh2) inhibits hair follicular morphogenesis and affects wound closure [96]. Cell proliferation, essential to regeneration and wound closure, is also strongly regulated by epigenetic enzymes that affect chromatin states. In the presence of an epidermal wound, hair follicular bulge stem cells (HFBSCs) are recruited to the site of injury to repair and close the wound and do so by generating TA cells [96]. Further, HFBSCs possesses the ability to differentiate into epidermal cells [96]. Therefore, DNA methylation and histone modifications together lend an “open” or “closed” accessibility configuration which influences the expression of genes involved in the wound healing process. Epigenetic-specific gene expression outcomes channelizes stem cells into different phenotypes based on the physiological cues received. However, regardless of the source of SCs, the transformation of SCs to TA cells pushing keratinocyte proliferation is a necessary step and is driven by p63 phosphorylation and induced expression [91].

Complex combinations of cellular and molecular mechanisms initiate wound healing and these processes are regulated by genetic and epigenetic events (Table 3). Lately, evidence implicating the influence of epigenetics in wound healing has been reported and occurs in all four phases of wound healing [19,97]. This includes early-response influenced by the injury and biochemical signals, inflammation, proliferation and migration of epithelia and wound closure [19,97]. Epigenetic events regulate the repair machinery at transcriptional and post-translational levels. Epigenetic events that strongly influence early healing stages are decreased global methylation through a reduction of histone H3 lysine 27 (H3K27) trimethylation, down-regulation of polycomb group and upregulation of histone demethylases [19]. Such epigenetic patterns increase the accessibility of the keratinocyte and fibroblast genomes to the transcriptome machinery, positively contributing to skin repair (Table 3) [98]. Contrary to a decrease in global histone methylation, a decrease in gene specific repressive H3K27 trimethylation enhances the inflammatory process by promoting interleukin-12 (IL-12) expression and has been seen in diabetic conditions where H3K27 demethylase, JmjC domain-containing protein 3 (Jmjd3) is inhibited [99].

Several factors contribute to a decrease in wound healing in individuals with diabetes. The pathophysiological conditions of the disease itself impairs several process and, with respect to dermal responses, include poor collagen accumulation, reduced epidermal barrier function and quantity of granulation tissue, decrease in keratinocyte and fibroblast migration and proliferation [100]. Molecular biomarkers contributing to the delay have been identified from biopsy samples from the epidermis and include c-myc overexpression, nuclear localization of β-catenin which prevent epithelization by hindering keratinocyte migration, hyper-proliferation and incomplete differentiation [101].

Wound healing is highly compromised in diabetes. A study using skin punch biopsies as a model of wound healing revealed that as a combination, increased sirtuin levels and a decrease in Class I HDACs enhance the expression of α- tubulin associated with increased histone H3 lysine 9 (H3K9) histone marks [102]. Such epigenetic combinations are found to enhance the proliferation and differentiation of keratinocytes fostering tissue repair and is nitric oxide (NO) dependent [103]. Alternatively, increase in histone acetyltransferases (HAT) such as P300/CBP-associated factor (PCAF) and GCN5 enhance wound healing through processes independent of NO [103]. Therefore, aberrations in HAT activity tend to slow down the repair process in skin, especially in diseased conditions such as diabetes.

Upon wound formation, epidermal keratinocytes release interleukin-1 (IL-1) that activate adjoining keratinocytes [8]. The activation process is governed by numerous factors and include growth factors and factors that assist the inflammation process. This process in turn incites the expression of specific keratin protein markers, keratins K6 and K16 [8,104]. Keratinocyte migration is enhanced by the keratins K6 and K16 devoid of changes in the intracellular architecture of the keratinocytes [105,106,107]. Currently, the mechanisms that contribute to the cessation of the activation cycle are not very well understood. However, feed-back inhibition of the activation process itself and corticosteroids are thought to regulate the inhibition of the activation signal [8]. Corticosteroids are believed to suppress K6/K16 expression and keratinocyte migration by the inhibition of the epidermal growth factor (EGF) [8].

**Table 3:** Epigenetic regulation of epidermal stratification in wound healing.

Epigenetic modification	Enzyme involved	Epigenetic Effect	Effect on Epidermal development	Reference
Methylation	DNMT1	Global hypermethylation	Maintenance of epidermal progenitor self-renewal capability	[108]
Histone methylation	Histone demethylase, JmjC domain-containing protein 3 (Jmjd3)	Demethylation of trimethylated histone H3 lysine 27 (H3K27me3)	Epidermal stratification, proliferation and differentiation	[19]
	Ubiquitously transcribed X chromosome (UTX)			
	Histone methyltransferase SET domain containing 8 (SETD8)	Histone H4 lysine 20 (H4K20) mono-methylation	–	[108]
Histone acetylation	Histone deacetylase 1/2	Global histone acetylation; H3 acetylation; P38 activation	Promote proliferation and differentiation of epidermal stem cells	[109]
Polycomb repressive complex 1 (PCR1) and polycomb repressive complex 2 (PCR2)	Enhancer of zeste 1 (Ezh1) and Enhancer of zeste 2 (Ezh2)	Trimethylation of histone H3 lysine 27 (H3K27)	Maintains stem cell quiescence regulates epidermal differentiation and stratification	[99,110]

In skin, the IFE regulate skin homeostasis and injury repair [3]. The IFE stem cells maintain the population of cells in the IFE and exhibit both replicative potentials and differentiation capabilities. Several models explaining the homeostasis of IFE have been theorized they are (1) Discreet epidermal proliferative units that are present in IFE with a single SC to about 10 TA cells; (2) A single progenitor population existing to contribute to IFE homeostasis; (3) a mixed population of SCs and TA cells each with distinct functions [3]. The specific population that plays a role in tissue regeneration upon injury is still not clear. In the majority of the cases, SCs are recruited to the site of injury to begin the repair process. However, further investigations are necessary to validate the involvement of SCs as sole agents of wound repair. Experiments conducted in animal models using the labeling technique showed that wound repair of the tail epidermis in these animals is largely due to SCs [111]. However, further analysis revealed that the expanding population of cells from the SCs expressed very high levels of p63 and were in fact TA cells [112].

Cancer metastasis bears close similarity to the wound healing process. Basal cell carcinomas (BCCs) are promoted through deregulation of Hedgehog (Hh) signaling [113,114]. Studies have shown that SCs from the hair follicle bulge expressing activated smoothened (Smo) down-regulate Hh contributing to BCC [115]. This appears similar to the wounding process where hair follicular stem cells (HFSCs) are recruited to the wound site and, when Hh signaling is down-regulated, gives rise to BCC-like tumors [116].

## 6. Conclusions

Epigenetic manipulation of ESCs demostrates much potential for therapy in skin-related disorders as well as in wound healing and skin cancers such as melanomas. Although a majority of studies focus on individual effects of epigenetic mechanisms in ESC regulation that affect stemness and plasticity, most often it is the cumulative interplay of epigenetic signatures that regulates the expression of genes involved in ESC self-renewable capabilities and proliferation capacities. Using current technological advances in the field of epigenetics, the collective influences of epigenetic combinations in ESC homeostasis, stemness, plasticity and cues for epidermal stratification which are yet not clear need to be deciphered.

## Abbreviations

AP1: Activator protein-1; AP2: activator protein 2; AJ: Adheren junctions; BM: Basement membrane; BCC: Basal cell carcinoma; C/EBPs: CCAAT/enhancer binding protein; Ctip2: Chicken ovalbumin upstream promoter transcription factor-interacting proteins 2; DNMT: DNA methyltransferase; DNMT1: DNA methyltransferase 1; E-cadherin: Epithelial cadherin; ESC: Epidermal stem cells; ECM: Extracellular matrix; Ezh1: Enhancer of zeste 1; Ezh2: Enhancer of zeste 2; EGF: Epidermal growth factor; FOXM1: Forkhead box protein M1; GFP: Green fluorescent protein; HF: Hair follicle; HFBSC: hair follicular bulge stem cells; HFSC: Hair follicle stem cells; Hh: Hedgehog; HAT: Histone acetyltransferase; HDAC: Histone deacetylase; H3K9: Histone H3 lysine 9; H3K27: Histone H3 lysine 27; H4K20: Histone H4 lysine 20; HMTs: histone methyltransferases; IFE: Interfollicular epithelium; Jmjd3: JmjC domain-containing protein 3; Klfs: Krüppel-like family of transcription factors; MCSP: Melanoma chondroitin sulfate proteoglycan; Lrg1: Leucine-rich α-2-glycoproein 1; miRNA: microRNA; PCAF: P300/CBP-associated factor; P-cadherin: Placental cadherin; PKC: protein kinase C; PLAG1: pleomorphic adenoma gene 1; PCR1: Polycomb repressive complex 1; PCR2: Polycomb repressive complex 2; PPARs: Peroxisome proliferator-activated receptors; SETD8: SET domain containing (lysine methyltransferase) 8; SG: Sebaceous gland; Smo: Smoothened; SC: Stem cells; TA: Transit amplifying; TGF: Transforming growth factor; UTX: Ubiquitously transcribed X chromosome.
